# “This Is Not Our Disease”: A Qualitative Study of Influencers of COVID-19 Preventive Behaviours in Nguenyyiel Refugee Camp (Gambella, Ethiopia)

**DOI:** 10.3389/fpubh.2021.723474

**Published:** 2022-01-04

**Authors:** Ademe Tsegaye, Calistus Wilunda, Fabio Manenti, Matteo Bottechia, Michele D'Alessandro, Giovanni Putoto, Andrea Atzori, Daniel Frehun, Gabriel Cardona-Fox

**Affiliations:** ^1^Doctors With Africa CUAMM, Addis Ababa, Ethiopia; ^2^African Population and Health Research Center, Nairobi, Kenya; ^3^Doctors With Africa CUAMM, Padova, Italy; ^4^Doctors With Africa CUAMM, Gambella, Ethiopia; ^5^School of Advanced International Studies (SAIS) Europe, Bologna Institute for Policy Research (BIPR), Johns Hopkins University, Bologna, Italy

**Keywords:** COVID-19, refugee camp, refugees, SBCC, Gambella, Nguenyyiel, Ethiopia

## Abstract

The COVID-19 pandemic has infected more than 263 million people and claimed the lives of over 5 million people worldwide. Refugees living in camp settings are particularly vulnerable to infection because of the difficulty implementing preventive measures and lack of medical resources. However, very little is known about the factors that influence the behavioural response of refugees towards COVID-19. There is an urgent need for field evidence to inform the design and implementation of a robust social and behaviour change communication strategy to respond to the threat posed by COVID-19 in humanitarian settings. This study examines factors influencing COVID-19-related behavioural decisions in the Nguenyyiel refugee camp located in Gambella, Ethiopia using data collected from focus group discussions and key informant interviews in September 2020. The evidence suggests that while a number of factors have been facilitating the adoption of COVID-19 prevention measures, including good general knowledge about the virus and the necessary preventive strategies and the active engagement by community leaders and non-governmental organisations, important structural and cultural factors have hindered the uptake of COVID-19 prevention measures. These include: difficultly staying at home to minimise physical contact; overcrowding in the camp and within home dwellings; a lack of hand sanitizers and masks and of funds to purchase these; inconsistent use of facemasks when available; COVID-19 denial and misconceptions about the disease, and other cultural beliefs and habits. Overall, the study found that refugees perceived COVID-19 to pose a low threat (susceptibility and severity) and had mixed beliefs about the efficacy of preventive behaviours. This study identified gaps in the existing information education and communication strategy, including a lack of consistency, inadequate messaging, and a limited use of communication channels. While awareness of COVID-19 is a necessary first step, it is not sufficient to increase adoption of prevention measures in this setting. The current communication strategy should move beyond awareness raising and emphasise the threat posed by COVID-19 especially among the most vulnerable members of the camp population. This should be accompanied by increased community support and attention to other barriers and incentives to preventive behaviours.

## Introduction

Since the WHO declared a Public Health Emergency in January 2020, COVID-19 has infected more than 260 million people and claimed the lives of almost 5.2 million people worldwide by 26th November 2021 (1). Ethiopia has reported close to 271,000 cases and 6,700 deaths (1), although these numbers are likely to represent gross underestimations (1). Refugees and IDPs have been particularly hard hit. Those living in camp settings, most of whom are in middle-income countries (LMICs), are particularly vulnerable to infection because of overcrowding and a general lack of access to water, sanitation and hygiene (WASH) services, and medical resources.

The COVID-19 pandemic has created a global health crisis unprecedented since the outbreak of the Spanish Flu a century ago. Until the recent development of successful vaccines, the only means to protect oneself and prevent the spread of the virus was through the strict observation of social distancing, quarantines, lockdowns, and the promotion of basic preventive measures, like frequent hand washing, self-isolation, and the use of face masks in public. These measures are particularly difficult to observe in marginalised and over-crowed refugee camp settings, where access to facemasks, water, soap, heath facilities, and proper healthcare is limited.

At the outbreak of the pandemic, experts form UNHCR and IOM described refugee camps as “ticking time bombs waiting to explode,” and warned that COVID-19 could have a devastating effect and spread like “wild fire” if it reached the world's largest refugee camps. There are over 26 million refugees in the world, about a third of whom live in camps. An additional 48 million people are internally displaced as a result of conflict and human rights violations (2). This population includes more than a million Rohingya refugees in Bangladesh, 3.6 million Syrian refugees in Turkey, 289,000 in Iraq, 232,000 South Sudanese in Uganda and 189,000 in Kenya (3).

Having fled violence and social collapse, this population is already highly vulnerable. Refugees live in precarious and overcrowded conditions, suffer from a high prevalence of physical and mental health problems, and are constantly facing the risk of deportation and an uncertain future. Their access to health care is limited due to financial, administrative, legal, and language barriers (4). Most LMICs that host the overwhelming majority of refugees and IDPs lack enough vaccines, COVID-19 tests, hospital beds, intensive care units, or ventilators to treat their own populations.

As of the time of publication, no large outbreaks of COVID-19 have been reported in refugee or IDP camps. This may be partially explained by the fact that cases in many LMICs that host refugees have yet to peak and many refugee camps are located in isolated places. It is also very likely that COVID-19 has taken hold in some camps, but that the virus has been under-reported due to a lack of testing, and fear of stigmatisation on the part of the refugees. The refugee populations also tend to be relatively young, which affords a certain degree of protection. A modelling study of the potential impact of a COVID-19 outbreak in a Rohingya refugee camp in the Kutupalong-Balukhali area of Bangladesh, for example, projected that an outbreak would be characterised by a high transmission but a low death rate. Even if refugee camp residents manage to avoid getting ill as a result of COVID-19, the pandemic is likely to take a tremendous toll on these vulnerable populations.

With over 814,535 refugees and IDPs, Ethiopia is host to one of the largest forced migrant populations in the world. According to UNHCR, 368,822 of them (45.3%) are refugees that have fled the conflict in South Sudan (5). Approximately 82,614 of these refugees reside in the Nguenyyiel Refugee Camp, in Ethiopia's Gambella region (6).

In refugee camps and regions with fragile health systems, the range of medical and epidemiological responses to emerging disease outbreaks can fall short, as was demonstrated by the response to the Ebola outbreak in West Africa (7). In these contexts community-based responses are critical to preventing the spread of infections.

Community-based responses, however, are affected by local perceptions of the threats posed by the disease and of the efficacy of the proposed protection measures. The perceptions of threat are a combination of people's perceived susceptibility to infection and perceived severity of the disease. Perceived efficacy, on the other hand, represents a combination of *response efficacy*, or the perceived effectiveness of the recommended response to avoid the threat, and perceived *self-efficacy*, or people's belief in their own ability to adequately perform the recommended response. Both concepts are derived from the extended parallel process model of Social and Behaviour Change Communication (SBCC) for emergency preparedness. This model is recommended for emergency situations because it takes into account the increased risk perception that populations are likely to experience as a result of an emergency (8). An effective SBCC strategy design must also take into account the various levels of influence that interact to influence behaviour. The Social Ecological Model, informed by Bronfenbrenner's 1979s seminal work (9), recognises four levels of influence: (1) individual; (2) family and peer networks; (3) community; and (4) social/structural levels (10). Ideally, a messaging strategy should be evidence-based, respond to misinformation present within the community, and induce rational, adaptive, and protective behaviours in the population. In humanitarian settings, however, very little is usually known about the complex interplay among the changing epidemiology, media coverage, the measures put in place to control the epidemic, the public's perception of risk, and their behaviours. Considering this, the design and implementation of a robust SBCC response to COVID-19 in humanitarian settings needs to be informed by evidence generated through action research. West Africa's experience with Ebola clearly illustrated that SBCC interventions were critical in changing harmful health practises that were contributing to the rapid spread of Ebola (11).

Unpublished preliminary reports produced by community outreach agents showed evidence of a significant “knowledge-action gap” with regards to COVID-19 in the Nguenyyiel Refugee Camp (12). While camp residents were aware of the existence of COVID-19, they perceived it to be a low risk. The disease was mostly seen as a “*Ferenj*” affair, or a disease that only affects white people and to which Black Africans are immune. It became increasingly apparent that humanitarian agencies' communication strategies that had focused exclusively on COVID-19's modes of transmission and means of prevention were not bringing about the desired behavioural changes. Crowded camp conditions and a lack of infrastructure also presented significant challenges to the implementation of COVID-19 preventive measures and the adoption of health recommendations. This study was conducted to generate evidence to inform the design of more effective communication strategies that could bring about the desired behavioural changes to prevent the transmission of COVD-19 in the camp.

More specifically, this study sought to explore factors that influence refugees' response to COVID-19 at the different levels—individual, family, community, and social/structural—as identified by the Social Ecological Model. The study also sought to identify the perceived threats and efficacy beliefs that influence responses to prevention and control of COVID-19 and to identify opportunities for actions in the camp. The evidence generated would help to develop and implement appropriate messages to support specific interventions targeting all camp residents including vulnerable groups to adopt prevention measures. The evidence would also contribute to the development of appropriate SBCC messages tailored to the existing local community-based organisations such as youth clubs, self-help mothers' associations, mutual help associations, sport and religious groups, to support the COVID-19 response.

## Methods

The Nguenyyiel Refugee Camp, located in Itang Woreda, is the largest and newest camp in Ethiopia's Gambella region (13). The camp is home to 82,744 South Sudanese refugees, all of them from the Nuer tribe (13). It is divided into four zones, each with about 20,000 people. In total, there are 17,613 households, 51,150 children below age 15, and 14,281 women of reproductive age (14). As of the end of 2020, there were 13 reported COVID-19 cases and no deaths in the camp (14). The camp was selected for this study because Doctors With Africa Collegio Aspiranti e Medici Missionari (CUAMM) has been implementing a health project there providing medical services to the refugees specifically focusing on maternal and child health services.

This study was designed as a qualitative action research project using focus group discussions (FGDs) and key informant interviews (KIIs) with adult male and female populations of refugees residing in the Ngunyyiel Refugee Camp. Focus groups are used to explore views on health issues, programs, and interventions. The group interaction encourages respondents to explore and clarify individual and shared perspectives. This method was thus deemed most appropriate to answer our research objectives of exploring factors that influence refugees' response to COVID-19 and identifying the perceived threats and efficacy beliefs that influence their responses to COVID-19 prevention and control measures. KIIs were used to provide information from an expert's perspective to complement and triangulate the information from FGDs. All adult residents of the camp who were able to provide informed consent were eligible to participate in the study.

FGD participants were chosen through sampling techniques to guarantee fair representation according to geographic distribution throughout the camp, demographic indicators, and their social position in the camp. FGDs were conducted with the following groups: elderly men (aged > 60 years), elderly women (aged > 60 years), middle-aged men (aged 30–59 years), middle-aged women (aged 30–59 years), male youth (aged 18–29 years)„ female youth (aged 18–29 years), and members of the refugee central committee. The FGD participants were identified from the refugee register maintained by the UNHCR. Table 1 shows the makeup of the FGDs. The FGDs included 41 participants including 10 elderly women and men, 10 middle-aged men and women, 11 Refugee Central Committee (RCC) members and 10 male and female youth. The RCC is the main decision making and coordination body for refugees in the camp. Most of the participants (41.5%) had 1–8 years of schooling and about a third had no formal education, with the elderly being more likely to be non-educated. Most (73.2%) had stayed in the camp for 4–5 years.

**Table 1:** Characteristics of participants of focus group discussions.

**Characteristics**	**Elderly men and women (*n* = 10)**	**Middle-aged men and women (*n* = 10)**	**RCC members** **(*n* = 11)**	**Male and female youth (*n* = 10)**	**Total (*n* = 41)**
Age^*^, Median (min–max)	65 (61–70)	36 (33–49)	41.5 (38–45)	23 (18–28)	40 (18–70)
**Sex**, ***n*** **(%)**					
Female	5 (50)	5 (50)	5 (45.5)	5 (50)	20 (48.9)
Male	5 (50)	5 (50)	6 (54.6)	5 (50)	21 (51.2)
**Education level**, ***n*** **(%)**					
None	7 (70)	4 (40)	3 (27.3)	0 (0)	14 (34.2)
Grade 1–8	3 (30)	3 (30)	6 (54.6)	5 (50)	17 (41.5)
Above grade 9	0 (0)	3 (30)	2 (18.2)	5 (50)	10 (24.4)
**Duration in the camp**, ***n*** **(%)**					
2–3 years	1 (10)	2 (20)	5 (45.5)	3 (30)	11 (26.8)
4–5 years	9 (90)	8 (80)	6 (54.6)	7 (70)	30 (73.2)

* *Data on age were missing for five Refugee Central Committee (RCC) members*.

Participants of KIIs included community leaders and humanitarian organisation staff members with knowledge of COVID-19 prevention and response. KIIs were conducted with three NGO staff involved in SBCC activities and three community leaders in the camp.

Data was collected in September 2020 by trained field interviewers (facilitators) using open-ended KIIs and FGD interview guides. The interview guides were written in English and translated into the Nuer language in which the interviews were conducted. All KII and FGD sessions were audio recorded with the consent of participants, and field notes were taken to document non-verbal cues. FGDs were conducted in venues close to respondents' houses while KIIs were conducted in respondents' offices or homes.

Two teams, each composed of three field interviewers fluent in Nuer and English, collected data. A field supervisor supervised the teams. A team composed of female field interviewers facilitated women's FGD sessions while a team composed of male interviewers facilitated FGDs involving men.

All audio recordings were transcribed verbatim, translated to English, and uploaded into *Atlas.ti* for inductive coding and thematic analysis. After reading through the transcripts, the research team members developed and reviewed a coding framework. Data were then summarised under four broad themes, namely: (1) Factors facilitating prevention of COVID-19; (2) factors hindering COVID-19 prevention; (3) unintended consequences of COVID-19 prevention measures; and (4) SBCC interventions. Lower-level themes were presented under each broad theme and pieced together to provide an overview of the content relating to that specific theme. Quotes from respondents were selected to represent a typical response or to illustrate a deviant opinion.

Ethical approval for the study was provided by the Gambella Region Health Bureau Ethics Review Committee and The Agency for Returnees and Refugees Affairs (ARRA). All participants in this study provided verbal informed consent.

## Results

The FGDs and KIIs explored a few key themes. The section below discusses the factors that facilitated and that hindered the uptake of COVID-19 preventive measures among the refugee population living in the camp. In both cases, a distinction is made between factors affecting behaviour at the individual, household, and community/social levels. The study then proceeds to identify refugees' perceived threats and efficacy beliefs that influenced their behaviour towards the pandemic. Finally, it explores the measures taken in the camp and some unintended consequences produced by these measures.

## Factors Facilitating the Uptake of COVID-19 Prevention Measures

### Awareness About COVID-19 (Individual Level)

Refugees in the camp, for the most part, exhibited a high level of awareness and knowledge about COVID-19. Participants felt that they had received sufficient information about the virus, and they attributed this to the sensitisation work that humanitarian organisations had been conducting in the camp since the outbreak of the pandemic.

“*As my colleagues explained it more, we have been made aware by many institutions about the symptoms of coronavirus which include fever, cough, diarrhoea, sneezing.”* FGD, Elderly Men

“*The information given to us about COVID is enough. All NGOs are working hard to teach us on how we can protect ourselves from coronavirus.”* FGD, Middle-aged women

As a result of the sensitisation campaigns, COVID-19 was a common topic of conversation within the camp. Participants believed that the problem in the camp was not a lack of awareness about the virus, but that people were failing to take preventive measures.

“*We discuss about COVID at home. This coronavirus has dominated all the dialogues at homes and on streets.”* FGD, Elderly men

“*I don't think there is a problem on awareness. The problem is taking the preventive measures.”* KII, NGO staff

Participants showed a high level of awareness about the measures that needed to be taken if they or others began exhibiting symptoms of COVID-19. Among the measures mentioned were visiting the health facility, self-isolating at home, and staying away from symptomatic individuals.

“*Everyone who feels sick should go to the closest health facilities whenever they have some symptoms. No one should stay at home while sick.”* FGD, Elderly men

“*If a person has a cough, she/he can't sleep together with other persons. If a child has fever, other individuals should distance themselves from the child”* FGD, Elderly women

Participants also showed a high level of knowledge about COVID-19 prevention strategies. Most were able to recall with ease the recommended public health strategies including hand washing, social distancing, avoiding handshakes, staying away from symptomatic individuals, using face masks, and not sharing them with others.

“*We have to keep our social distance; we should wear face masks and we should avoid handshakes.” FGD, Male youth*

“*COVID-19 preventive measures are important things like avoiding handshakes, keeping social distance and staying far from sick persons.”* FGD, RCC Men

“*We have learned many things. We use soap, we maintain our distance, we stay far from the person who has fever, we stay far from the person who has a cough, we stay far from a person who has a common cold and far from persons who sneeze; the masks should be worn by only one person. Masks should not be used by two or more people even among family members.”* FGD, Elderly men

Respondents had a generally good understanding about the means through which COVID-19 is transmitted from person to person.

“*We heard that air droplet transmission occurs when a person is in close contact with an infected person and exposure to potentially infective respiratory droplets occurs, for example, through coughing, sneezing or very close personal contact resulting in the transfer of air droplet through the mouth and the nose.”* FGD, Elderly men

“*We know that coronavirus transmits from person to person through coughing and sneezing.”* FGD, Male youth

Participants were also aware that COVID-19 could sometimes result in death, and that there was no known treatment for the disease.

“*We are taking care of coronavirus because we have heard that there is no treatment for it.”* FGD, Elderly men

“*This disease is very dangerous. There is no treatment for it.”* FGD, Female youth

There were, however, some misconceptions and inaccurate information about the severity of COVID-19. Some respondents, for example, believed that the disease killed instantly.

“*What we hear about coronavirus is that it kills persons immediately after infection.”* FGD, RCC men

“*It kills people immediately after it has reached the person's lung.”* FGD, RCC men

### Communication Within Households (Household Level)

COVID-19 prevention messaging was clearly spreading within households in the camp. The study revealed that men were the primary recipients of the information, because they were also the ones most likely to venture out of the home and to become exposed to health messages. They, in turn, transmitted this information to the women in the home and to other family members.

“*I told to my wife emphasizing that children can do a lot to keep themselves and others safe. I introduce the idea of social distancing, standing further away from friends, avoiding large crowds, not touching people.”* FGD, Elderly men

“*Even though I don't get information about COVID, my husband will tell me what he has heard. Men do not sit at home like women and you have to follow what they say.”* FGD, Middle-aged women

### Role of Community Leaders and Humanitarian NGOs (Community/Social Level)

Community leaders played a key role in promoting COVID-19 prevention measures in the camp. They raised awareness about COVID-19 when food was distributed in the camp, in the streets using megaphones, and during house to house visits and public gatherings in churches and schools. Community leaders also provided refugees with the means to wash their hands, ensuring that people adhered to social distancing guidelines when in public, for example, while waiting in food distribution lines. They also acted as critical links between the community, the authorities, and the NGOs.

“*We pass the information to the community during food distribution. We arrange water for washing; we ensure their hands are washed. We also maintain social distance during food distribution time. We report the inadequacy of resource allocation to the bodies concerned.”* FGD, RCC men

*They visit our homes and tell us not to share many things with our neighbours, not to share drinking cups even with home members, and not to share eating utensils with our neighbours.”* FGD, Elderly men

“*The community leader has told us day and night to wash our hands with soap and water and to keep social distance.”* FGD, Elderly women

Participants expressed a high level of trust in the NGOs that were involved in raising awareness about COVID-19 and that were promoting preventive measures. They particularly recognised the work that OXFAM was doing.

“*I trust the international non-governmental organization who brought us here, I know they are working hard for us and they will bring masks.”* FGD, RCC men

“*The OXFAM plays a great role to provide us training, provide us water, soap, and increase our awareness to fight against the diseases. They provide us places for hand washing, carry out campaigns, and provide information using megaphones.”* FGD, Elderly men

NGOs working in the camp (particularly OXFAM), had ensured that water and soap were available for the people to wash their hands.

“*The use of soap and water has been a very good thing and the soap has been available here in the camp; you can see people washing their hands with soap and water along the roads and even at home people are doing it.”* FGD, Elderly men

### Preventive Measures Taken (Individual Level)

FGD participants mentioned several measures that they were taking to prevent COVID. These included washing hands with soap, changing clothes after venturing outside of the house, properly disposing of used tissue paper, using coughing/sneezing etiquette, avoiding handshakes, avoiding symptomatic individuals, and exercising social distancing.

“*First of all, we wash our hands with soap and water. We ask guests to wash their hands before coming into our house.”* FGD, RCC men

“*We cover our nose and mouth while sneezing or coughing with a flexed elbow. We don't throw used tissue papers everywhere.”* FGD, Elderly men

“*If I need to cough, I have to turn to the side to avoid the transmission of the disease and also I don't greet friends by shaking hands.”* FGD, Elderly women

## Factors Hindering the Uptake of COVID-19 Prevention Measures

Despite having a basic understanding about COVID-19 and the measures that should be taken to prevent infection, refugees living in the camp were not always able or willing to follow preventive guidelines consistently. Discussions with community participants and key informants showed increasing evidence of a “knowledge-practise” gap.

### Individual Level Factors

Individuals had difficulty observing social distancing recommendations and limiting physical contact with others because of the need to venture out of the home in search of sources of livelihoods and because of a strong tradition of visiting relatives.

“*People who are working among us get only 800 Ethiopian Birr (US$21). This money is not enough to bring food to our family; it should be supplemented by wild foods. We are on the run; I cannot close my door and stay at home. We are suffering with hunger so we can't stay home and starve.”* FGD, RCC men

“*Yeah, we will spend our time everywhere because here in this camp we are visiting our relatives on daily basis.”* FGD, Female youth

The camp residents claimed that they lacked money to buy COVID-19 supplies such as face masks and hand sanitizers.

“*We have no money. Only a few people have this mask; these two things are not available.”* FGD, middle-aged women

“*The refugees don't have money to buy soap and face mask. They are not wearing face masks because they don't have them.”* KII, Humanitarian NGO staff

When available, face masks were used inconsistently. Individuals tended to use facemasks only in situations where they were mandatory, for example, when riding public transportation.

“*I have seen little change recently; I have seen masks worn by women when they were the first people who refused masks. They were forced to wear masks before getting into buses to go to Gambella.”* FGD, RCC men

Although masks were available at local shops, the majority of the camp's residents could not afford to buy them. A lack of hand sanitizers also meant that most people had to wash their hands with soap and water to maintain hand hygiene.

“*How many people are there in your home? You mean all your family can wear the same mask? It may be only you who can afford to buy a mask. Some people cannot afford to buy masks for all family members.”* FGD, RCC men

“*We don't have enough facemask, and we don't have sanitizers. We try to use them but it's challenging.”* FGD, Women

### Overcrowding at Home (Household-Level Factors)

Families live in crowded conditions because of large family sizes and inadequate housing. The camp is overcrowded making it very difficult for residents to observe social distancing and creating potentially risky conditions for the spread of infectious diseases.

“*When the number of families and relatives increase, people start to congest in the same house.”* FGD, Middle-aged women

“*You may find five to ten people sleeping in the same house.”* FGD, RCC men

### Misconceptions About COVID

There were a few shared misconceptions about COVID-19 that were responsible for hindering the uptake of preventive measures. The most common one, perhaps, was the belief that COVID-19 was a disease that only affected white people, and that Africans living in warmer climates were somehow immune to it.

“*Some people say: I'm sorry you are believing white people. Just leave it. This is not our disease. But I would tell them that this disease is for everyone.”* FGD, Middle-aged women

“*There are people who say this disease is only for white people and it can't be transmitted to black people.”* FGD, Women

“*Some people say COVID-19 does not affect the people living in hot environments, which is not true. Some people in the beginning were saying COVID-19 could not affect black people.”* KII, Humanitarian NGO staff

There were also shared misconceptions about preventive measures and cures. For example, some believed that drinking alcohol and smoking cigarettes could prevent infection.

“*When you hear the young men talking, they will say things like: why are you not drinking wine or smoking cigarettes? If you drink, you will not be infected by the Corona virus.” FGD, Male youth*

Some individuals ignored COVID-19 prevention guidelines despite being aware of what they were supposed to do.

“*… some people don't understand things easily, they ignore the disease even if it is considerably evident.”* FGD, RCC men

“*…of course, the community knows how to prevent COVID-19. Nevertheless, they don't apply the preventive strategy. They are ignoring the presence of COVID-19.”* KII, Humanitarian NGO staff

At one extreme, some camp residents denied the existence of COVID-19 all together.

“*When trying to tell people about COVID, they never understand saying this is false, this is a false thing.”* FGD, RCC men

“*…people don't accept the presence of the disease called COVID-19”* KII, Humanitarian NGO staff

Others failed to follow the guidelines because they believed that COVID-19 was not particularly severe as evidenced by the very small number of reported cases and deaths in the camp.

“*The reason they ignore the disease is that they have heard of people recovering very quickly of coronavirus. They don't believe the disease is serious enough to kill people.”* KII, Humanitarian NGO staff

Those who denied the existence of the virus did so mainly because they had never seen someone who was sick or had died from COVID-19.

“*People ask questions like: if it is real, let me see persons who are affected by coronavirus. If coronavirus is really true, I have to see real patients.”* FGD, RCC men

### Perceived Low Severity of COVID-19 (Individual)

Overall, camp residents did not perceive the threat of COVID-19 to be particularly severe. This is because the camp was not particularly affected.

“*The community does not believe that the disease is serious enough to kill people. They ignore the disease considering it not that serious.”* KII, Humanitarian NGO staff

Individuals attempting to observe COVID-19 prevention measures where often under pressure not to do so by those who denied the existence of the virus.

“*We are forced to shake hands with those who ignore this disease saying: this is false; there is no disease.”* FGD, RCC men

“*People are discouraged by others. If they see you wearing the mask, they may say ‘why are you wearing this mask?' There is no coronavirus.” FGD, Elderly women*

“*When you keep your distance, they will challenge you saying wrong words and we try to tell them about COVID, but they don't believe the disease exists.”* FGD, Female youth

### Culture and Tradition (Community/Society-Level Factors)

The study identified a number of social and cultural factors particular to Nuer society, which hindered the uptake the recommended protective measures.

Social gatherings such as small group meetings, communal meals, worshipping and working together, are a central part of the Nuer culture. Observing social distancing guidelines in this context thus proved to be particularly challenging. During social gatherings, COVID-19 prevention measures are often ignored.

“*It is difficult to prevent Nuer form sitting and gathering together. If you go to the market you see many people sitting together.”* FGD, Female youth

“*According to our tradition, we should be together, sit together, eat together and work together.”* FGD, male youth.

The Nuer believe that eating together is unavoidable. Eating alone, or not sharing food is seen as a sign of selfishness.

“*Nuer people share things together; when a person refuses to share food in order to avoid COVID, he should be called selfish.” FGD, RCC men*

Because of high unemployment rates among the refugees, the issue of boredom and idleness also presents a challenge. Unemployed refugees prefer to spend their time meeting with friends and relatives to chat or play games.

“*We the refugees feel that staying at home alone is boring, so every man including me spend our time at our local cafeterias; We drink coffee and tea there. We share some conversation about our country there. We listen to some news from our country and sometimes it brings pleasure.”* FGD, Elderly men

“*But how can we spend our days? We sit together and talk.”* FGD, Elderly men

Avoiding the habit of shaking hands as a form of greeting one another has been particularly difficult. Handshaking is an integral part of Nuer culture. It was considered inappropriate or impolite to avoid handshakes.

“*Nuer culture is one of several serious factors affecting the prevention of COVID-19. In Nuer culture, they love shaking hands with others. The people promote handshakes. It is offensive not to shake hands as a greeting. A Nuer person does not allow covering of nose with something.”* FGD, RCC men

“*There are many things in Nuer culture which influence the prevention of coronavirus, one of them is handshakes…”* FGD, RCC men

Avoiding a handshake as a preventive measure, in fact, was considered rude and as something that could lead to conflict in the community.

“*Coronavirus prevention causes conflict in our community. When trying to avoid shaking hands with a person from a rural area, they feel something bad. These are not good for Nuer culture.”* FGD, RCC men

The Nuer tradition of visiting relatives and people who are ill also presented a risk for viral transmission of COVID-19. Preventing people from visiting a sick person and having physical contact with that person was seen as unacceptable.

“*For example, my neighbour's son was sick yesterday. I visited him today early in the morning before coming to work. My visit is not only to see him and turn my back without touching. He may condemn me if I don't touch him. To avoid being condemned by my neighbour, I touched him. I don't know if the disease was COVID.”* FGD, RCC men

Participants found it very difficult to require guests to wash their hands when visiting their homes.

“*When a guest comes home and you give him an order to wash his hands before sitting down; saying ‘go there and wash your hands,' it can bring conflict. I don't feel comfortable telling people to wash their hands.”* FGD, RCC men

Another challenge to the uptake of the recommended protective measures, derived from Nuer culture, was the presence of traditional healers in the camp who claimed to be able to treat COVID-19 using traditional medicine.

“*Yes, there are traditional healers here in the camp.”* FGD, RCC men

“*Yes, there are traditional healers for corona; they treat cough and chest pain. I know some traditional medicines among plant roots including ‘wangmach,' and ‘chiok buoka.' These are good medicine for Corona.”* FGD, RCC men

“*If you have a cough, there are healers. You will go to them and they will give you the medicines from the forest and you will be healed.”* FGD, Female youth

Some participants recognised that a certain sense of fatalism, inherent in Nuer culture, may also have hindered, to some extent, the uptake of preventive measures in the camp. The Nuer considered death to be a normal thing that should not be feared. This means that people are not too afraid of dying of COVID-19 and are therefore not very likely to adopt preventive measures.

“*Death is not new, and you can't occupy the soil after death. These kind of sayings are most influential in discouraging people from taking preventive measures.”* FGD, RCC men

“*If a person is trying to take preventive measures, other Nuer will say death is not new thing.”* FGD, RCC men

Finally, participants noted that, for the large part, children in the camp were not observing any COVID-19 prevention measures.

“*Children are not stoppable, while a person may try to keep himself at home; the children are impossible to keep at home.”* FGD, RCC men

## Perceived Threats and Efficacy Beliefs That Influence the Response to COVID-19

### Perceived Threat

Initially, people did not feel threatened because they did not believe that COVID-19 was real. After a number of cases were reported in other refugee camps in Gambella, some individuals began feeling susceptible to infection.

“*We didn't believe that the disease was real. But when coronavirus reached the camp, all of us became afraid and understood that what we were told was true.”* FGD, Elderly men

“*When we were given an announcement that coronavirus had come, we did not believe it, but when it was announced that it affected people here in the camp it caught our attention that this disease was for real.”* FGD, Middle-aged women

Despite a number of confirmed cases, some camp residents continued to believe that COVID-19 was a white people's disease that could not harm people living in warm regions. These individuals did not view COVID-19 as a threat.

Many minimised the severity of the disease, likening it to other common illnesses present in the community. They did not believe they had any good reason to fear COVID-19.

“*The community does not believe that the disease is serious enough to kill the people. They ignore the disease considering it not that serious.”* KII, Humanitarian NGO staff

“*Old people are saying COVID is simple. It is like common cold or malaria. They don't take it as a serious disease.”* FGD, Male youth.

This was mainly because death from COVID-19 was rare in the camp.

“*When people see that it is not killing a lot of people, they say that it is not a serious disease.”* KII, Humanitarian NGO staff.

Some, however, did believe that COVID-19 was a severe disease that could result in death.

“*This disease is very dangerous. There is no treatment for it.”* FGD, Female youth

“*We understand that coronavirus can kill people.”* FGD, Elderly men.

### Efficacy Beliefs

Overall, FGD participants accepted that the COVID-19 prevention methods being promoted, including frequent hand washing, the use of face masks in public, and social distancing, were effective in preventing transmission.

“*We understand that masks can help prevent the spread of the virus from the person wearing the mask to others, but we don't have enough masks.”* FGD, Elderly men

“*If you shake hands with everyone you will transmit the disease. If you wear facemask even though you have a cough, there is no way to transmit the disease.”* FGD, Female youth

There were, however, a mixture of self-efficacy beliefs. Some FGD participants felt that COVID-19 had been brought about by God to punish humanity. These participants believed that prevention measures were futile, and that only God could protect them.

“*I suspect that this disease is brought to us by God through air. God wants to punish his people. God should have brought the poison to the earth through something like air.”* FGD, Elderly men

“*We will not succeed in prevention by ourselves; we will be protected by God.”* FGD, RCC Men

Others expressed low self-efficacy to prevent infection because they lacked the recommended protection supplies.

“*It is difficult for us to prevent the disease. We have no prevention equipment.”* FGD, Elderly men

Overall, most FGDs participants felt that they were capable of taking preventive measures to protect themselves against COVID-19. This sense of confidence derived in part from their wartime experience, which they had survived, and which they saw as significantly more threatening than COVID-19. They felt that protecting themselves from this virus in the camp would be easier than surviving the violence in their own country.

“*It is a dangerous disease. If we are responsible enough to protect our health, we can prevent it. It will not be as difficult as preventing deaths from war. Millions of people have died in South Sudan due to war. COVID is not difficult like war. We can prevent the disease.”* FGD, Elderly women

Surviving violence in South Sudan could also result in the adoption of a cavalier attitude towards COVID-19 within the camp.

“*We are victims of war that occurred in South Sudan. We sometimes have trauma. You can see many people here don't care about serious things.”* FGD, Elderly men

### Additional Consequences of COVID-19 Prevention Measures

An added consequence of the promotion of COVID prevention measures has been the improvement of sanitary conditions in the refugee camp.

“*No hand washing practice before this disease, now people are well aware. Being clean in your area, avoid eating un-boiled food. This disease has improved sanitary awareness in the family*” FGD, RCC men

“*I wake up in the morning and start to clean the home, and my wife, child and the whole family join me in cleaning the home. We also clean the place of my cattle; now my children have learned that, and they now wake up before me. They started cleaning their houses, they reach the outside and move on to other places of my home.”* FGD, Elderly men.

### Social and Behaviour Change Communication Interventions

Humanitarian organisations principally made use of megaphones and loudspeakers to broadcast IEC messages in the camp. Community leaders also delivered IEC messages via house-to-house visits. Respondents, however, recognised that this method also increased the risk of transmitting the disease. They believed that there was a need for radio broadcasting IEC messages.

“*Currently, community leaders walk house to house to deliver health information. However, walking by feet is not good because there may be contact. We need a radio station here in the camp to pass information to everybody in the camp.”* FGD, RCC men

“*OXFAM provides information to the whole camp using megaphones mounted on a vehicle. It provides information to the whole camp. We need these kinds of things in this camp.”* FGD, RCC men

“*the way health messages are delivered to us is through the use of megaphone, loudspeakers and so on in the language of the communities by health care workers.”* FGD, Female youth

Posters were also displayed in the marketplaces to convey COVID-related messages.

“*There are also mass awareness raising activity at the community level using megaphones and loudspeakers. There are also a lot of key message posted around market centres. The community can easily look and identify what has to be done to prevent COVID-19.”* KII, Humanitarian NGO staff

Some individuals first became aware of COVID-19 after attending training sessions in the camp.

“*Our awareness has improved; we attended so many trainings and learned about COVID.”* FGD, Elderly men

IEC messaging in the camp focused principally on people's need to observe social distancing, avoid shaking hands, wear masks, wash hands frequently, and avoid individuals exhibiting flu-like symptoms.

“*We have been told that there is disease and there is no need for handshakes, and we have been told to wear masks. We have been told also to stay clean. All home members should frequently wash their hands. These are the ways to protect ourselves.”* FGD, RCC men

“*If you are giving your kids food, you have to wash your hand, if you are going to the market to buy something you have to use a mask and you cannot greet anyone on the road and if there is anyone who greets you, you have to say hi without touching his/her hand. This is the information we are given”* FGD, Elderly women

“*…so, we are told that people cannot sit in one place or be together, we have to wash our hand with soap. If you see someone coughing or having common cold, you should not be close to her/him. The third thing is we have to wear a face mask*.” FGD, Middle-aged women

Respondents identified a number of gaps in the existing IEC messaging. They thought, for example, that IEC activities were conducted very sporadically, giving people the impression that COVID-19 was no longer a threat.

“*In this community everyone knows coronavirus. However, the IEC activities are not continuously conducted. If there is no follow up from us, we may think that coronavirus does not exist.”* FGD, RCC men

With regards to the content of the messages broadcasted, participants believed that the severity of COVID-19 was not being emphasised well enough. This was leading camp residents to believe that COVID-19 was not very severe, and to relax the observation preventive measures.

“*They don't have that much promotion regarding the seriousness of the disease. Different partners are focusing on raising awareness about COVID. Most of the IEC activities focus on awareness raising activities not giving emphasis on seriousness of the disease.”* KII, Humanitarian NGO staff

“*The severity of the disease is not reported by different organizations especially in the Gambella region. So, the community understands that there is no death from COVID-19 and are not serious enough to take preventive measures.”* KII, Humanitarian NGO staff

“*The community has some information regarding COVID-19, but the seriousness of COVID-19 is not given much emphasis.”* KII, Humanitarian NGO staff

There was also a general perception that the IEC delivery methods were largely ineffective in reaching everyone, especially those who stay at home. As previously noted, women who stay at home tended to receive their information regarding COVID-19 from their husbands who ventured outside of the home.

“*The people who have information about COVID are the ones who attend churches and go to the market areas. Those people who are sitting at home are not getting adequate information about COVID-19.”* FGD, Male youth

Participants believed that existing IEC activities could be made more effective with the use of more pictures and symbols. They also believed that more extensive use could be made of existing social networks as well of existing community, administrative, and religious structures.

“*It is good to show pictures to people showing the cause of the disease, picture of infected persons and picture of someone who takes preventive measures.”* FGD, RCC men

“*It is good to establish community social network to improve the communication system. If we have such network, once the information reaches to COAs, it can easily pass to the subsequent community structures.”* KII, Humanitarian NGO staff

Camp residents were most likely to trust information conveyed by health workers, community and religious leaders, NGO staff, and people who speak the same language as they do.

“*In my view, we trust you because we have the same language and the same traditional way of life. When I was in Dima the humanitarian staffs didn't provide us the real information like you do now. The life in Dima was difficult. Now we enjoy this camp because we get services from the people who share the same language and the same historical background with us.”* FGD, Elderly men

“*The one who will encourage me to take preventive actions are the people who work in the community because they are teaching us in the community, and those humanitarian workers, including RCC leaders, zonal leaders, block leaders, and all the people who have knowledge about this disease. They encourage me to take action.”* FGD, Female youth

“*Yeah, in my view, the community trusts the church leaders. Any information that pass through the church is accepted by the community.”* KII, Humanitarian NGO staff

## Discussion

This study found that refugees in the Nguenyyel Camp in Gambella, Ethiopia had good general awareness about COVID-19, the strategies they needed to follow to prevent contagion, and the measures they needed to take when and if symptoms occurred. Community leaders and NGOs have been actively transmitting this information within the community and this knowledge has been passed along within households. The uptake of preventive behaviours has also been facilitated by greater availability of water and soap, and a greater awareness of the importance of frequent hand washing.

The study also found that important factors were hindering the uptake of COVID-19 prevention measures. These included some structural factors, such as overcrowded living conditions, challenges practising social distancing by staying at home, lack of money to purchase hand sanitizer and facemasks, and inconsistent use of facemasks.

The uptake of effective prevention measures was also hindered by a general low severity perception of the disease, particularly in light of other safety risks faced by refugees, cultural obstacles to social distancing, and the acceptance of death as a normal event that is out of people's control.

Overall, the participants had a low risk perception of the threat posed by the COVID-19 virus. Their responses showed that they did not believe that they were very susceptible to the disease or that the disease was very severe. These perceptions were clearly reinforced by the fact that the virus has not impacted the camp very severely.

Participants also showed a mixture of efficacy beliefs. While most believed that the prevention measures promoted in the camp were indeed effective (response efficacy), some participants felt that there was not much they could personally do to prevent from becoming infected (self efficacy).

House-to-house visits by volunteers and use of megaphones and loudspeakers were the most common methods for the dissemination COVID-related IEC messages in the camp. These messages were focused primarily on promoting social distancing, mask wearing, and frequent handwashing. This study, however, identified a number of gaps in the existing IEC strategy. These included a lack of consistency in awareness raising, inadequate content, particularly with regards to the severity of the disease, and limited use of available communication channels.

Our study's findings are generally consistent with the findings of other studies of refugee populations living in camp settings. A study conducted on South Sudanese and Congolese refugees in Uganda showed that most refugees and asylum seekers were well-informed about COVID-19 and about the measures they needed to take to protect themselves from infection (15). For the most part, the refugee population in Uganda was following the proscribed COVID-19 measures but also experienced some difficulty practising social distancing, staying at home, and wearing facemasks. This study also showed evidence of the spread of misinformation and misconceptions about contracting, avoiding, and treating COVID-19. Like in Ethiopia, a common misconception was that the virus affected only whites and foreigners and that black Africans were immune. One of the limitations of the Ugandan study, however, was that it only included community leaders, so it is unclear whether ordinary members of the community shared the same perceptions.

Another study of Syrian refugees living in Turkey showed a very high level of COVID-19 awareness (16). Unlike the refugees in Nguenyyiel Camp, however, refugees in Turkey had access to television, social media, and the internet as sources of information about COVID-19, which facilitated the diffusion of IEC. Despite a high level of awareness, community members became less inclined to adhere to the proscribed preventive measures overtime as a type of lockdown fatigue settled in. As in Nguenyyiel Camp, some refugees in Turkey denied the existence of COVID-19 and minimised the risk it posed to them (16).

## Conclusion

The study in Gambella shows that while general knowledge about COVID-19 is a necessary first step to prevent the spread of infection, it does not guarantee the uptake of preventive measures within the community. Both significant structural and cultural challenges to the adoption of preventive measures must also be addressed. Further emphasis should be placed on the susceptibility and severity of COVID-19, particularly among the most vulnerable members of the population such as the elderly and individuals with underlying health conditions. Self-efficacy should be further encouraged. A comprehensive SBCC strategy must address the widespread misconceptions and the cultural factors that are hindering the adoption of preventive measures.

Additional material support to the community, making available face masks and hand sanitizer, largely out of reach to refugees, can also go a long way in promoting the adoption of preventive behaviours.

This qualitative study offers an initial yet valuable insight into the prevailing community perceptions related to COVID-19 within a refugee camp setting. The findings outlined in this study applied to all groups targeted. Within our sample, we did not note any major differences across demographic groups with regards to knowledge of the virus or risk adversity. A more in-depth quantitative inquiry that looks at a larger sample may further identify the factors that best predict community adherence to the recommended preventive measures. In light of this study's findings, we have proposed a SBCC communication strategy (Appendix 1), and have developed a list of key messages to educate the refugee population and counter some prevailing myths on COVID-19 (Appendix 2).

## Data Availability Statement

The raw data supporting the conclusions of this article will be made available by the authors, without undue reservation.

## Ethics Statement

The studies involving human participants were reviewed and approved by Gambella Region Health Bureau Ethics Review Committee. The Agency for Returnees and Refugees Affairs (ARRA) also approved the study. Participants provided verbal informed consent. Written informed consent for participation was not required for this study in accordance with the national legislation and the institutional requirements.

## Author Contributions

AT, FM, MB, GP, MD'A, and AA contributed to conception of the study. AT, CW, and GP contributed to design of the study. AT, DF, and MB facilitated the collection of and transcription of the data. AT and CW analysed and wrote the first draft of the study report. FM and GP reviewed the first draft report. GC-F wrote the first draft of the manuscript. All authors contributed to the manuscript revision, read, and approved the submitted version.

## Funding

This project was funded by a grant from UNICEF.

## Conflict of Interest

The authors declare that the research was conducted in the absence of any commercial or financial relationships that could be construed as a potential conflict of interest.

## Publisher's Note

All claims expressed in this article are solely those of the authors and do not necessarily represent those of their affiliated organizations, or those of the publisher, the editors and the reviewers. Any product that may be evaluated in this article, or claim that may be made by its manufacturer, is not guaranteed or endorsed by the publisher.
