# Supplementary material for: “This Is Not Our Disease”: A Qualitative Study of Influencers of COVID-19 Preventive Behaviours in Nguenyyiel Refugee Camp (Gambella, Ethiopia)
Source: Front Public Health. 2022 Jan 4;9:723474. doi: 10.3389/fpubh.2021.723474 (PMC8764300; doi:10.3389/fpubh.2021.723474)
Supplement: Supplementary file 1 [file Data_Sheet_1.PDF]

Appendix: COVID-19 Overview and Key messages

Coronavirus Disease (COVID-19)

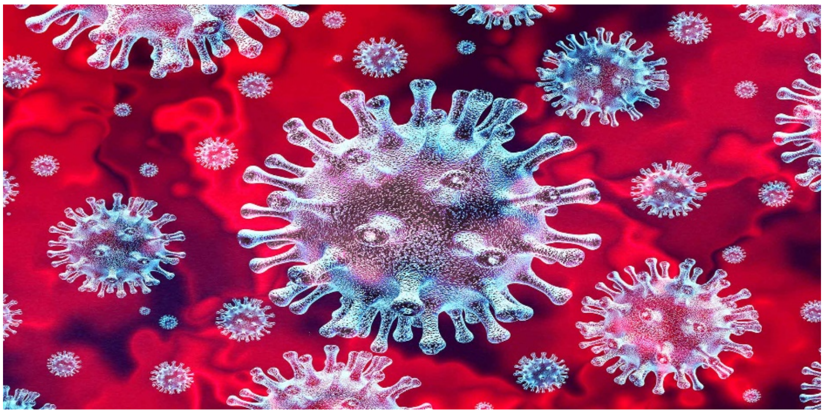

OVERVIEW AND MYTH BUSTERS

DOCTORS WITH AFRICA CUAMM

WHAT IS COVID-19?	
COVID-19 is a disease caused by a new coronavirus<br><br>It is a virus that causes respiratory illnesses	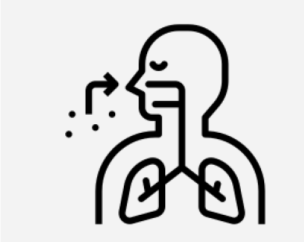

WHAT ARE THE SYMPTOMS?	
Fever	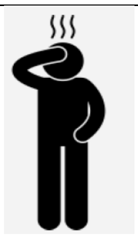
Cough	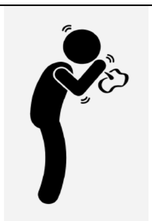
Shortness of breath	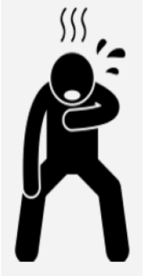
If you are ill with the above symptoms report to the nearby health facility	

WHO IS AT RISK?	
People aged 60 years and over	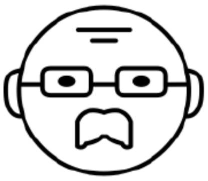
those with underlying medical problems like high blood pressure, heart and lung problems, diabetes, obesity or cancer	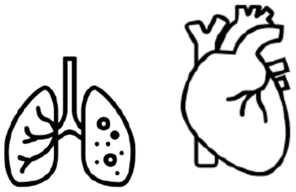
**However, anyone can get sick with COVID-19 and become seriously ill or die at any age.**	

HOW DOES COVID-19 SPREAD?	
Sick person to healthy person; cough, sneeze or talking droplets	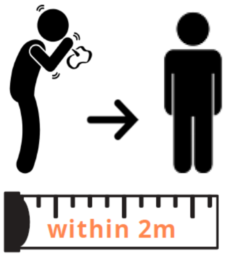
Someone who doesn't seem sick to a healthy person; cough, sneeze or talking	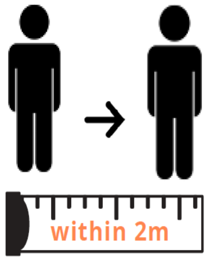
Contaminated surface when a sick person touches a surface and then a healthy person touches the same object	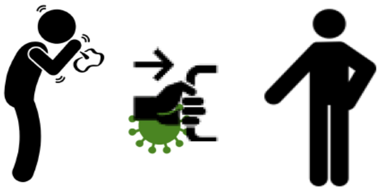

HOW TO PROTECT YOURSELF?	
Wash hands with soap / detergent for 20 seconds with moving water	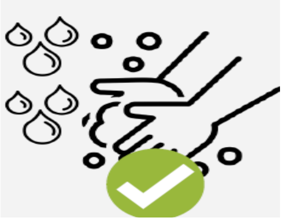
Put on face masks	
Avoid touching your face	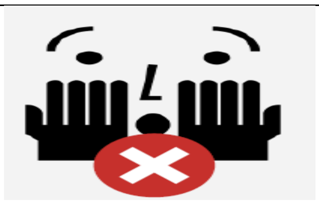
Stay 2 metres away	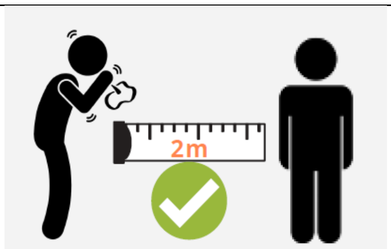
Avoid surfaces that could be contaminated	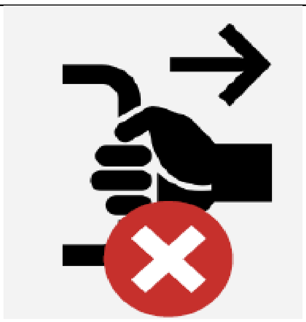

HOW TO PROTECT YOURSELF? (CONT.)	
Avoid crowds and gatherings<br><br>Avoid places where people are close together<br><br>Avoid high traffic areas	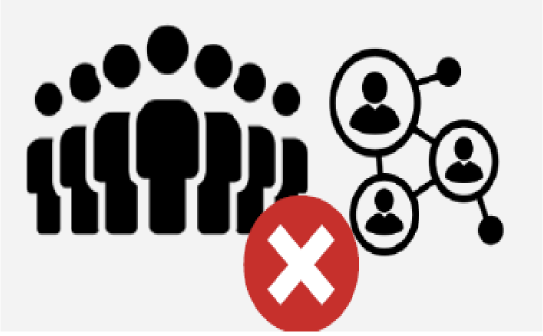
Avoid hand shake instead<br><br>Wave, tow touch, bow	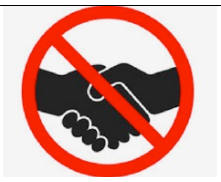<br>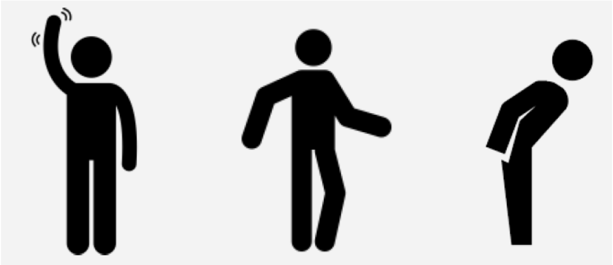

Key Messages

- Coronavirus virus can spread in hot environments
- COVID-19 affects people of all races without distinction
- So far in the world more than 1.7 million people have died of COVID-19
- In Ethiopia more than 100,000 people have been infected with COVID-19 of which more than 1,800 have died
- In Gambella region more than 996 people are infected with COVID-19
- If you get seriously ill with COVID-19, the treatment cost is not affordable.
- The treatment for complicated COIVD-19 is neither affordable nor accessible in remote areas of the country.
- People can be asymptomatic but still transmit the disease.
- Wash hands with soap / detergent for 20 seconds with moving water before getting into a house and after touching sick people.
- COVID-19 is a deadly disease!
- If you love your family, relatives and friends, reduce your contact at this difficult time.
- The prudent sees danger and hides himself, but the simple go on and suffer for it!
